# Serum Albumin and Uric Acid Levels in Hypertensive Patients: A Cross-Sectional Analysis From Central Tamil Nadu, South India

**DOI:** 10.7759/cureus.76766

**Published:** 2025-01-01

**Authors:** Rock B Dharmaraj, Kirubakaran Thangavel, Vijayapriya Indirajith, Neethu George, Ganesan Subramanyan, Balaji S Mahendran, Vibhulagavan Gnanamoorthy, Neeraj V Mohandas, Vijay Anand V, Meera George

**Affiliations:** 1 Community Medicine, Dhanalakshmi Srinivasan Medical College and Hospital, Siruvachur, Perambalur, IND; 2 Biochemistry, Dhanalakshmi Srinivasan Medical College and Hospital, Siruvachur, Perambalur, IND; 3 Community Medicine, Saveetha Medical College & Hospital, Saveetha Institute of Medical and Technical Sciences, Chennai, IND; 4 Community Medicine, Family Health Centre, Kumily, IND

**Keywords:** albumin level, association, hypertension and therapy, metabolic risk factors, observational cross-sectional study, serum uric acid level

## Abstract

Introduction

Hypertension represents a significant global health challenge, with an increasing incidence among adults. Despite the prominence of infectious diseases, non-communicable conditions like hypertension remain a silent yet critical health concern. Liver and kidney functions play crucial roles in blood pressure maintenance, with serum albumin and uric acid serving as key metabolic indicators.

Objective

The primary objective of this study is to analyze the association between serum albumin and uric acid levels in hypertensive patients aged 30-50 years. The secondary objective is to determine whether deranged serum albumin and uric acid levels are associated with other variables like body mass index and blood pressure values.

Methods

An analytical cross-sectional study was conducted between September and October 2021 at a hospital in Perambalur, Tamil Nadu, India. The study employed non-probability convenient sampling to recruit hypertensive patients aged 30-50 years. Participants with coronary artery disease, stroke, liver disease, renal failure, hyperuricemia, gout, diabetes mellitus, or taking medications that affect albumin or uric acid levels were excluded. Blood pressure measurements were taken after ensuring adequate rest, and 4 ml of venous blood was collected from each participant for biochemical analysis. Serum albumin and uric acid levels were determined using the analyzer. The data analysis was performed using Microsoft Excel and statistical software. The statistical significance of the findings was evaluated using appropriate statistical tests, providing a robust framework for understanding the metabolic associations in hypertension.

Results

The study population of 150 hypertensive patients demonstrated a majority of 88 (58.67%) aged over 40 years and 62 (41.33%) under 40 years. The gender distribution revealed 87 (58%) males and 63 (42% females). The mean systolic blood pressure was 158.2 mmHg, with a mean diastolic blood pressure of 94.73 mmHg, indicating moderate to severe hypertension. Biochemical analysis showed an average serum uric acid level of 6.41 mg/dL and a mean serum albumin level of 3.54 mg/dL. Statistical analysis revealed a significant association between elevated uric acid levels and decreased serum albumin levels (p < 0.05), suggesting a potential interrelationship between these metabolic markers in hypertensive patients.

Conclusion

The study establishes hyperuricemia and hypoalbuminemia as significant risk factors for hypertension development or pathogenesis. Early detection of these metabolic derangements may provide opportunities for preventive interventions and potential disease management strategies. The findings emphasize the importance of comprehensive biochemical assessment in understanding and mitigating hypertension risk.

## Introduction

High blood pressure (hypertension) remains a critical yet often overlooked health concern in today’s fast-paced society. While we actively address various forms of social and professional pressures, this silent cardiovascular threat continues to pose significant health risks to the global population. The incidence of hypertension [1] among adults is showing an increasing trend these days. In contrast to infectious diseases, hypertension manifests its’ complications after a long latent period. The focus on infectious diseases has overshadowed hypertension and other chronic conditions, leading to their unchecked rise as critical public health concerns. According to the ‘iceberg phenomenon’, there are a lot of subclinical cases of hypertension present in the community. If we target those subclinical cases and also prevent the incidence of hypertension, there will be a great reduction in disease burden and cost spent for the management of complications [2] that will develop in the future. Therefore, early diagnosis of hypertension is crucial at this time. The liver and kidney play a major role in the maintenance of normal blood pressure [3]. Any derangements in their function alter the blood pressure. Serum albumin secreted by the liver is the major plasma protein that maintains colloidal osmotic pressure. It binds and transports cations, water, fatty acids, hormones, bilirubin, thyroxine, and pharmaceuticals [4]. Albumin is not only a scavenger for free radicals but also a reservoir for nitric oxide [5].

A decrease in albumin may reduce the reservoir of nitric oxide and increase the risk of free radical-induced endothelial damage. This results in vasoconstriction and may predispose to the development of hypertension [6]. Serum uric acid is formed from purine degradation and is primarily excreted through the kidney. Its normal serum level is maintained by a balance between the breakdown of purines and the rate of uric acid excretion. Elevated serum uric acid levels are associated with renal hypoperfusion, which may activate the renin-angiotensin-aldosterone system (RAAS). Uric acid also causes endothelial damage and vascular inflammation, which again activates the RAAS [7]. Thus, uric acid induces acute vasoconstriction via RAAS and contributes to the development of hypertension.

Clinical studies done in countries like Bangladesh [8], China [9], Korea [10], and Japan [6,11,12] concluded that patients with elevated uric acid levels and decreased albumin levels had a high chance of developing hypertension. It is imperative to focus on risk factors contributing to the development of hypertension. Among these, uric acid and albumin levels represent critical metabolic parameters that warrant comprehensive investigation to enhance prophylactic understanding. By systematically examining these biochemical markers, researchers can uncover valuable insights into the complex pathogenesis of hypertension, potentially enabling early detection and targeted preventive strategies [13]. The primary objective of this study is to analyze the association between serum albumin and uric acid levels in hypertensive patients aged 30-50 years. The secondary objective is to determine whether deranged serum albumin and uric acid levels are associated with other variables like body mass index and blood pressure values.

## Materials and methods

Sample selection

The study population comprised newly or recently diagnosed hypertensive patients aged 30-50 years. This analytical, cross-sectional study was conducted in the Department of Biochemistry during September and October 2021. Participants were recruited from the Department of General Medicine at the tertiary medical college hospital, in Tamil Nadu, India, and blood samples were collected and analyzed in the Central Laboratories using convenient non-probability sampling. Consent for treatment and open access publication was obtained or waived by all participants in this study. Institutional Ethics Committee of Dhanalakshmi Srinivasan Medical College Hospital issued approval (IECHS/IRCHS/DSMCH/NO 36-202).

The sample size was calculated using the formula N = Z²1-α/2 * p * (1 - p)/d², following the methodology described by Peter Bjornstad et al. [14]. With a prevalence of elevated uric acid levels among newly diagnosed hypertensive patients at 37.4% (p = 0.374), a two-tailed probability for 95% confidence interval (Z1-α/2 = 1.96), and an allowable precision of 7.75% (d = 0.0775), the calculated sample size was 150 participants.

Data collection

One hundred fifty newly or recently diagnosed hypertensive patients were recruited from the General Medicine Department of the tertiary medical college hospital. Hypertension was defined as systolic blood pressure >or= 140 mmHg and diastolic blood pressure >or= 90 mmHg. All study subjects were informed about the study aims and objectives. Written informed consent was obtained from each patient before participating in the study. The patient’s age, sex, body weight, and height were recorded. Body mass index (BMI) was calculated as weight in kg divided by height in meters squared from the data collected. Blood pressure was measured in all subjects by trained professionals using a sphygmomanometer or digital blood pressure machine. Blood pressure was measured on the left arm three times in the sitting position and supine position after the patient took a 10-minute rest. The first blood pressure measurement was discarded to avoid possible effects of anxiety. The average value of the second and third blood pressure measurements was counted for systolic and diastolic blood pressure. The patients were advised to avoid smoking, coffee, and tea for 30 minutes before blood pressure measurements.

Then patients were taken to the central laboratory in the tertiary medical college hospital for sample collection. 4 ml of venous blood sample was collected from each patient by trained professionals. The serum is separated from the blood sample, and the separated serum is used for the analysis of uric acid and albumin. Serum albumin and serum uric acid were estimated using the automated analyzer Erba XL 640 with an automated analyzer kit. All laboratory tests were done by trained professionals. Strict internal quality control was maintained in the laboratory for all investigations.

Study variables

The study examined several independent variables, including demographic factors (age and sex), biochemical parameters (serum uric acid (adult males: 3.4-7.0 mg/dL, adult females: 2.4-6.0 mg/dL) and albumin (3.5-5.0 g/dL) levels), and anthropometric measurements (BMI). The primary dependent variable was hypertension status, which was defined according to standard clinical criteria as having a systolic blood pressure greater than or equal to 140 mmHg and/or a diastolic blood pressure greater than or equal to 90 mmHg.

Data analysis

Data analysis was conducted using a comprehensive statistical approach, leveraging Microsoft Excel for initial data entry and SPSS software for advanced statistical processing. Categorical variables were systematically presented through frequency and percentage distributions, and numerical variables underwent descriptive statistical analysis, reporting mean and standard deviation. Statistical significance was rigorously assessed through multiple analytical techniques, including chi-square tests for categorical variable associations, independent t-tests for comparing means between groups, and correlation analyses to explore relationships between variables.

## Results

Baseline characteristics

The study population comprised 150 hypertensive patients. Based on age distribution, 88 (58.67%) patients were above 40 years of age, while 62 (41.33%) patients were 40 years or younger. The study had 87 (58%) males and 63 (42% females). The mean height among the subjects was 155.7 (± 4.29 cm), ranging from 148 to 170 cm. The mean weight among the subjects was 56.27 (± 4.73) kg, ranging from 40 to 70 kg. The mean BMI among the subjects calculated from height and weight was 23.16 (± 1.71) kg/m^2^, ranging from 18.2 to 27.8 kg/m^2^.

Blood pressure distribution among the study subjects

Among 150 hypertensive study subjects, the mean systolic blood pressure was 158.2 (± 11.99) mmHg, ranging from 140 to 200 mmHg. The mean diastolic blood pressure among the subjects was 94.73 (± 8.72) mmHg, ranging from 70 to 130 mmHg.

Distribution of uric acid and albumin among the study population: The mean serum uric acid among the subjects was 6.41 (± 1.6) mg/dL, ranging from 1.7 to 12.1 mg/dL. The mean serum albumin among the subjects was 3.54 (± 0.67) mg/dL, ranging from 1.7 to 5.6 mg/dL.

Distribution of serum albumin with serum uric acid in the study population

Considering the serum uric acid of the subjects with serum albumin distribution, 34 (55.73%) of the subjects with abnormal serum uric acid had abnormal serum albumin, which is higher compared to subjects with normal serum uric acid, of whom 35 (39.32%) had abnormal serum albumin, and the difference was statistically significant (p < 0.05). There is a significant association between abnormal uric acid levels and serum albumin levels in hypertensives (Table 1).

**Table 1:** Association between serum albumin and serum uric acid levels among study subjects. Chi-square test, table value: 3.92

Serum Uric Acid	Serum Albumin		Total	Chi-Square Value (p value)		
	Abnormal (decreased)	Normal				
Abnormal (increased)	34 (55.73%)	27 (44.26%)	61 (100%)	3.92 (0.048)		
					Normal	35 (39.32%)	54 (60.67%)	89 (100%)		
Total	69 (46%)	81 (54%)	150 (100%)							

Analysis of serum albumin distribution across BMI categories showed that subjects with normal BMI had abnormal serum albumin in 58 (44.3%) cases, which was lower compared to overweight subjects where 11 (57.9%) had abnormal serum albumin levels. This difference was not statistically significant (p > 0.05). When analyzing serum albumin distribution across systolic blood pressure ranges, abnormal serum albumin was most prevalent in the 140-159 mmHg range with 33 (50.0%) patients, followed by 160-179 mmHg range with 32 (43.8%) patients, and the least common in patients with ≥180 mmHg where 4 (36.4%) showed an abnormality. These differences were not statistically significant (p > 0.05). For diastolic blood pressure, abnormal serum albumin distribution was highest in patients with <90 mmHg where six (54.5%) showed abnormality, followed by 90-109 mmHg range with 59 (46.5%), and lowest in patients with ≥110 mmHg where four (33.3%) had abnormal levels. The difference in serum albumin distribution between different diastolic blood pressures was not statistically significant (p > 0.05) (Table 2).

**Table 2:** Distribution of subjects with different degrees of body mass index, systolic and diastolic blood pressure with normal and abnormal serum albumin levels.

		Serum Albumin				Chi-Square Value	p value
		Abnormal		Normal			
		Count	%	Count	%		
BMI	Normal	58	44.3	73	55.7	1.29	0.266
	Overweight	11	57.9	8	42.1		
Systolic Blood Pressure	140 – 159 mmHg	33	50.0	33	50.0	0.97	0.614
	160 - 179 mmHg	32	43.8	41	56.2		
	>= 180 mmHg	4	36.4	7	63.6		
Diastolic Blood Pressure	< 90 mmHg	6	54.5	5	45.5	1.11	0.574
	90 - 109 mmHg	59	46.5	68	53.5		
	>= 110 mmHg	4	33.3	8	66.7		

Among subjects with normal BMI, 56 (42.7%) had abnormal serum uric acid levels, which was higher compared to overweight subjects where five (26.3%) had abnormal levels; this difference was not statistically significant (p > 0.05). Analysis of serum uric acid distribution across systolic blood pressure ranges showed the highest prevalence in the 140-159 mmHg range with 33 (50.0%) patients, followed by the 160-179 mmHg range with 26 (35.6%), and the lowest in patients with ≥180 mmHg at 2 (18.2%). These differences were not statistically significant (p > 0.05). For diastolic blood pressure ranges, abnormal serum uric acid was most common in 90-109 mmHg group with 55 (43.3%), followed by <90 mmHg group with 4 (36.4%), and least common in ≥110 mmHg group with 2 (16.7%). The difference in serum uric acid distribution between different diastolic blood pressures was not statistically significant (p > 0.05) (Table 3).

**Table 3:** Distribution of subjects with different degrees of body mass index, systolic and diastolic blood pressure with normal and abnormal serum uric acid levels.

		Serum Uric Acid				Chi-Square Value	p value
		Abnormal		Normal			
		Count	%	Count	%		
BMI	Normal	56	42.7	75	57.3	1.86	0.173
	Overweight	5	26.3	14	73.7		
Systolic Blood Pressure	140 - 159 mmHg	33	50.0	33	50.0	5.46	0.065
	160 - 179 mmHg	26	35.6	47	64.4		
	>= 180 mmHg	2	18.2	9	81.8		
Diastolic Blood Pressure	< 90 mmHg	4	36.4	7	63.6	3.31	0.191
	90 - 109 mmHg	55	43.3	72	56.7		
	>= 110 mmHg	2	16.7	10	83.3		

## Discussion

The study, comprising 87 males and 63 females with newly and recently diagnosed hypertension, revealed a statistically significant association between elevated uric acid levels and decreased serum albumin levels (p=0.048). The analysis demonstrated that 34 subjects (22.6% of the study population) exhibited concurrent abnormalities in both parameters, specifically elevated serum uric acid and decreased serum albumin levels. Analysis of metabolic parameters revealed distinct patterns of biochemical alterations among hypertensive patients. Twenty-seven patients (18%) exhibited isolated elevation of serum uric acid with normal albumin levels, while 35 patients (23.3%) demonstrated decreased serum albumin with normal uric acid levels. The study identified that 61 patients (40.6%) showed abnormal uric acid levels, either independently or concurrent with albumin abnormalities. Similarly, 69 patients (46%) presented with abnormal serum albumin levels, either in isolation or combined with uric acid alterations. Notably, 96 patients (64%) demonstrated abnormalities in either one or both parameters. These comprehensive findings establish a significant pattern of metabolic dysregulation, suggesting the integral role of elevated serum uric acid and decreased serum albumin in hypertensive pathogenesis.

The study found that blood pressure measurements (both systolic and diastolic) and BMI categories showed no significant relationship with serum albumin or uric acid levels, whether normal or abnormal. The study established a clear relationship between elevated serum uric acid, decreased serum albumin, and hypertensive development. The pathophysiological mechanism involves uric acid-mediated activation of the RAAS leading to vasoconstriction, while decreased serum albumin induces vasoconstriction through oxidative stress pathways, both culminating in hypertension [15].

These findings have significant implications for both preventive and therapeutic approaches to hypertension management. From a primary prevention perspective, identifying hyperuricemia and hypoalbuminemia as risk factors enables targeted interventions before hypertension development. In secondary prevention, this understanding broadens therapeutic options by incorporating uric acid-lowering and albumin-elevating agents into conventional hypertension management protocols. This dual-level preventive approach offers a comprehensive framework for addressing hypertension through both prophylactic measures and enhanced treatment strategies.

A comparative study conducted on Bangladeshi hypertensive individuals demonstrated a positive correlation between elevated serum uric acid levels and hypertension development [8]. Their methodological approach focused exclusively on serum uric acid as a mediating factor, with participants stratified according to their uric acid levels. The findings revealed a higher prevalence of both hypertension and prehypertension in the cohort with elevated uric acid levels. This research, while more narrowly focused than the present study, provides corroborative evidence for the role of hyperuricemia in hypertensive pathogenesis. A large-scale cohort study conducted in the Kailuan Corporation among the Chinese population investigated the association between serum uric acid and hypertension. The study, initiated in 2006-2007, enrolled 39,233 adults who were stratified into four quartiles based on their serum uric acid levels. Following a four-year monitoring period concluding in 2011, 12,844 participants (31.31%) developed hypertension with an odds ratio of 1.17 [9].

A longitudinal study examined the relationship between hyperuricemia and hypertension over a seven-year period. At study initiation, hyperuricemia (≥6.8 mg/dL) was present in 25.6% of participants, with 18.7% exhibiting hypertension. The seven-year follow-up revealed hypertension development in 37.4% of participants and urinary albumin excretion in 18.0%. Statistical analysis demonstrated that elevated baseline serum uric acid levels increased the risk of incident hypertension, with a hazard ratio of 1.19 per 1 mg/dL increase in serum uric acid. Notably, this investigation focused on adolescents with type 2 diabetes mellitus, establishing that diabetes and obesity serve as additional contributory factors in hypertension development among adolescents with elevated serum uric acid levels [14].

The work by Bove et al. [16] on xanthine oxidase inhibitors provides compelling evidence for the therapeutic potential of targeting uric acid pathways in hypertension management, particularly in cases resistant to conventional treatments. While their interventional approach differs from the observational nature of the current study, both support the fundamental link between hyperuricemia and hypertension. The age-differential analysis among the Korean population [10] adds another layer of complexity, demonstrating stronger associations between hyperuricemia and incident hypertension in younger populations (under 55 years), with a quantifiable relative risk of 1.74 per 1.0 mg/dL increase in uric acid. The Japanese study [6] showed a 14% development rate of hypertension, markedly different from the 46% hypoalbuminemia rate observed in the current study. This disparity might be attributed to various factors, including population differences, study design variations, and the dynamic nature of serum albumin levels. Adding to this complexity, the Norwegian Oslo Health Study [17] presents an apparently contradictory finding, suggesting that increased serum albumin within the physiological range correlates with higher blood pressure. This seeming contradiction might be explained by a non-linear relationship between albumin and blood pressure or different pathophysiological mechanisms operating at various albumin levels.

The disparities observed between various studies on serum albumin, uric acid, and hypertension can be attributed to multiple complex factors that interplay in clinical research. Study design variations play a crucial role in these disparities; while some studies employ longitudinal cohort designs that track changes over time, others use cross-sectional or experimental approaches, each with their own inherent strengths and limitations. Methodological differences significantly impact study outcomes. For instance, the inclusion of recently diagnosed hypertensive patients who had been on antihypertensive medications for 1-2 months, despite this being an exclusion criterion, may have influenced the results. Such methodological variations, while sometimes unavoidable in real-world research settings, contribute to the disparities observed across different studies. Population characteristics also play a vital role; genetic variations, dietary patterns, and environmental factors specific to different geographical regions can significantly influence the relationship between serum markers and hypertension.

Strength

The study exhibits several significant methodological strengths that enhance its scientific rigor and clinical relevance. At its core, the investigation's focused examination of both serum uric acid and albumin levels in hypertension represents an innovative approach, distinguishing it from previous research that typically examined these parameters in isolation. The study did separate analyses of two distinct parameters, each providing insights into metabolic alterations in hypertensive patients The implementation of well-defined selection criteria created a homogeneous study population, which is crucial for maintaining internal validity and minimizing confounding factors. All analyses were performed in a single NABL (National Accreditation Board for Laboratories) accredited laboratory with standard operating procedures. These standardized laboratory protocols ensure that the observed associations between metabolic parameters and hypertension are based on accurate and precise data. The inclusion of both newly and recently diagnosed hypertensive patients provides valuable insights into early-stage disease processes, contributing to our understanding of pathogenic mechanisms.

Limitations

The study presents several important limitations that warrant careful consideration when interpreting its findings and planning future research. A significant constraint was the reliance on single-point measurements of biochemical parameters, which may not adequately capture the dynamic nature of these markers or account for potential temporal fluctuations. This limitation is particularly relevant for parameters like serum albumin and uric acid, which can vary due to multiple factors including diet, hydration status, and time of day. The COVID-19 pandemic imposed considerable restrictions on the research methodology, specifically limiting the ability to assess hypertension incidence in the general population, which could have provided valuable comparative data and enhanced the study's external validity. Additionally, while the study implemented specific exclusion criteria to maintain population homogeneity, the inclusion of some participants who had recently been on antihypertensive medications (within 1-2 months prior to the study) introduces a potential source of selection bias. These participants' prior medication exposure could have influenced their biochemical parameters and blood pressure readings, potentially confounding the observed associations.

Recommendations

**Figure 1:**
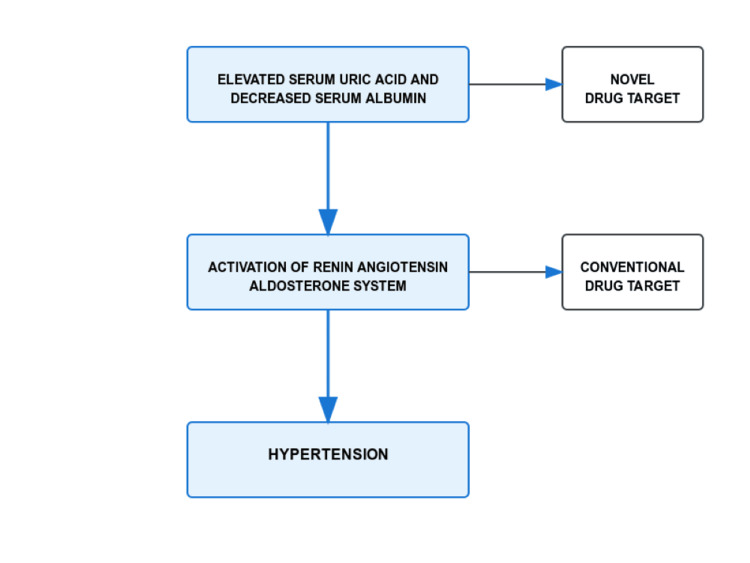
Future treatment modalities Kirubakaran T

Foremost, there is a pressing need for longitudinal investigations to establish temporal relationships between serum albumin, uric acid levels, and hypertension progression, with particular emphasis on pre-RAAS activation events across diverse ethnic populations. Multi-center collaborative studies across South India are essential to validate ethnic-specific cut-off values for these biomarkers in early hypertension risk assessment. Clinical practice should evolve to incorporate regular monitoring of these parameters as part of standard hypertension assessment protocols, especially in populations with high cardiovascular risk profiles. Treatment strategies should be broadened beyond traditional blood pressure control to address multiple pathophysiological pathways, including pre-RAAS mechanisms (Figure 1) [18]. Cost-effectiveness studies evaluating the economic impact of incorporating these biomarker assessments into routine clinical practice are also warranted.

## Conclusions

This cross-sectional analysis of newly and recently diagnosed hypertensive patients from South India has revealed significant insights into the metabolic basis of hypertension. This study revealed a distinct relationship between serum uric acid and serum albumin levels in hypertensive patients, with 55.73% of subjects having abnormal serum uric acid also showing abnormal serum albumin levels (p < 0.05). The study found that blood pressure measurements (both systolic and diastolic) and BMI categories showed no significant relationship with serum albumin or uric acid levels, whether normal or abnormal. Specifically, while there were variations in serum albumin levels across different BMI categories (44.3% abnormal in normal BMI vs 57.9% in overweight) and blood pressure ranges (50% abnormal in 140-159 mmHg systolic range), none of these associations reached statistical significance. Similarly, serum uric acid distributions across different blood pressure categories showed varying patterns but lacked statistical significance.
